# Correlation of Histopathological Profiles With Clinical and Radiological Risk Factors in Long-Bone Nonunion: A Prospective Cohort Study

**DOI:** 10.7759/cureus.91945

**Published:** 2025-09-09

**Authors:** Aqil Ahamed Mohideen Ahamed Sha, Uma Kiruthika Subramanian Lakshmi, Rafeek Ahmed M, Pragathesh Gowthaman Uma Mageswari, Shahul Hameed, Fadhil Mohammed

**Affiliations:** 1 Trauma and Orthopaedics, King's College Hospital National Health Service (NHS) Trust, London, GBR; 2 General Medicine, King's College Hospital National Health Service (NHS) Trust, London, GBR; 3 Orthopaedics, Dr. Somervell Memorial Church of South of India (CSI) Medical College and Hospital, Trivandrum, IND; 4 Critical Care Medicine, Christian Medical College, Vellore, IND; 5 Medicine and Surgery, Stanley Medical College and Hospital, Chennai, IND; 6 Internal Medicine, Stanley Medical College and Hospital, Chennai, IND

**Keywords:** bone necrosis, histopathology and microbiology, nonunion, prospective cohort, proximal humeral fracture

## Abstract

Background

Fracture nonunion is a significant complication of orthopedic trauma, affecting a notable proportion of long bone fractures and contributing to prolonged disability and increased healthcare costs. Although radiological classification into hypertrophic, atrophic, and oligotrophic types is traditionally used to estimate biological activity at the fracture site, imaging alone may not accurately predict healing potential. Histopathological evaluation offers direct insight into tissue viability, cellular activity, and infection, potentially enhancing prognostication and guiding treatment strategies.

Methods

This prospective cohort study included 80 patients with long bone nonunions who underwent revision surgery at Stanley Medical College and Hospital between March 2024 and March 2025. Clinical risk factors such as age, comorbidities, smoking history, and prior infections, along with demographic data and fracture characteristics, were recorded. Radiological classification was performed for each case, and intraoperative samples from the nonunion site were subjected to detailed histopathological and microbiological examination. Healing outcomes were assessed at six months postoperatively and correlated with clinical, radiological, and histopathological parameters using appropriate statistical tests.

Results

Among the 80 patients, the majority were men and sustained diaphyseal fractures. Diabetes and prior infections were the most common comorbidities, and both were significantly associated with necrotic bone histology and poorer healing outcomes. Radiologically atrophic nonunions often showed variable histological viability, with viable bone or fibrocartilage present in more than 60% of cases, highlighting the limitations of radiological assessment alone. Histopathology revealed a spectrum of findings: viable bone in 24 (30%) patients, fibrous/fibrocartilage in 20 (37.5%) patients, necrotic bone in 14 (17.5%) patients, and chronic inflammation in 12 (15%) patients. Viable bone histology was strongly associated with complete healing in 20 (83.3%) patients, whereas necrotic bone and chronic inflammation predicted persistent nonunion. Culture-positive nonunions showed significantly lower healing rates (four (28.6%)) compared to culture-negative cases (46 (69.7%)).

Conclusion

Histopathological evaluation provides critical biological insights beyond radiological classification and strongly correlates with healing outcomes in long bone nonunions. Viable bone histology predicts favorable healing, while necrotic bone, chronic inflammation, diabetes, and culture-positive infections are associated with poor outcomes. Integrating clinical, radiological, microbiological, and histopathological data can improve risk stratification and help identify patients who would benefit from biological augmentation in addition to mechanical stabilization. Larger multicenter studies are warranted to validate these findings and develop standardized algorithms for the evaluation and management of fracture nonunions.

## Introduction

Fracture nonunion is a serious complication in orthopedic trauma, frequently resulting in prolonged disability, impaired function, and increased healthcare costs [1]. It is commonly defined as the failure of a fracture to heal by nine months post-injury, combined with a lack of radiological or clinical progression over the preceding three months [1]. The incidence of nonunion varies by anatomical site, fracture severity, fixation method, and patient-related risk factors such as smoking, diabetes, infection, and poor vascularity, with rates reported between approximately 2% and 10% [2]. Based on radiographic appearance, nonunions are classified as hypertrophic, atrophic, or oligotrophic. Hypertrophic nonunions exhibit abundant callus formation-indicating preserved biological potential but insufficient stability, while atrophic nonunions show minimal or absent callus, suggestive of compromised vascularity and cellular activity; oligotrophic nonunions fall between these two extremes [1]. However, radiological classification alone may not accurately reflect the underlying biological status, and histopathological examination provides direct insights into tissue viability, cellular responses, vascularization, and infection - key factors influencing healing potential and therapeutic decision-making [3].

Histological evaluation of nonunion sites has documented a spectrum of tissue findings, including viable woven bone, fibrocartilage, fibrous tissue, necrotic bone, and chronic inflammatory infiltrates [3]. Correlating these histopathological patterns with demographic, clinical, and radiological variables may uncover predictors of poor outcomes and guide tailored management strategies - for instance, deciding when biological augmentation (e.g., bone grafting or growth factors) is warranted versus when mechanical stabilization alone is adequate [2]. Despite the importance of these correlations, relatively few studies integrate histopathological profiles with patient-specific risk factors and radiological classifications. The present study aims to address this gap by analyzing histological features at nonunion sites and correlating them with clinical and radiological risk factors, ultimately enhancing our understanding of the biological environment and guiding optimal management strategies.

The objective of this study was to analyze histopathological features at long bone nonunion sites and to correlate these findings with clinical risk factors, radiological classification, and microbiological culture results, in order to assess their predictive value for healing outcomes after revision surgery.

## Materials and methods

This study was designed as a prospective cohort study conducted in the Department of Orthopaedics, Government Stanley Medical College and Hospital, Chennai, from March 2024 to March 2025. Patients presenting with established nonunion of long-bone fractures requiring revision surgery were enrolled consecutively after obtaining informed consent. Nonunion was defined as the absence of clinical and radiological evidence of progressive healing for at least six months following the initial fracture. The study protocol was reviewed and approved by the Institutional Ethics Committee.

A total of 80 patients fulfilling the inclusion criteria were included in the study. Patients aged 18 years and above with nonunion of long bones (humerus, radius, ulna, femur, tibia), confirmed both clinically and radiologically and who were planned for revision surgery and in whom adequate bone and tissue samples could be obtained from the nonunion site, were included. Patients with pathological fractures due to malignancy, those with insufficient sample size for histopathological analysis, and patients unwilling to participate in the study were excluded.

For each patient, demographic data such as age, gender, occupation, and comorbidities including diabetes mellitus, hypertension, smoking, alcohol intake, and steroid use were recorded. Fracture characteristics including site, side, mechanism of injury, type of fracture (open or closed), Gustilo-Anderson classification for open fractures, and initial treatment modality were documented. Clinical examination at presentation, history of prior infections, and time elapsed since the initial injury were also noted.

Radiological evaluation was performed using plain radiographs in at least two orthogonal views. Each nonunion was classified as hypertrophic, atrophic, or oligotrophic based on standard criteria. The Radiographic Union Scale for Tibial fractures (RUST) score was also documented wherever applicable. Evidence of implant failure, bone loss, or sequestrum was noted.

During revision surgery, representative bone and soft tissue samples were collected from the nonunion site under sterile conditions. All specimens were divided for histopathological examination and microbiological culture. Histopathological evaluation was carried out by a pathologist blinded to clinical and radiological findings, using hematoxylin and eosin-stained sections. These findings were categorized into viable bone with osteoblastic activity, fibrocartilage, fibrous tissue predominance, necrotic bone, or chronic inflammatory changes. Microbiological cultures were processed for aerobic and anaerobic organisms to identify the associated infections. All patients were followed up at regular intervals post-operatively, and clinical as well as radiological assessment for fracture healing was performed at each visit. The final healing status was documented at six months after revision surgery and was categorized as complete healing, partial healing, or persistent nonunion based on standardized radiological and clinical criteria.

Data were entered into Microsoft Excel (Microsoft, Redmond, WA) and analyzed using SPSS software version 25 (IBM Corp, Armonk, NY). Continuous variables were expressed as mean and standard deviation, and categorical variables as frequencies and percentages. Correlation between histopathological findings and clinical or radiological parameters was analyzed using Chi-square or Fisher’s exact test for categorical data and independent t-test or Mann-Whitney U test for continuous data. Multivariate logistic regression was performed to identify predictors of adverse histopathological findings, and a p-value less than 0.05 was considered statistically significant.

## Results

The study population comprised 80 patients with a mean age of 42.5 years. In the study population, 52 patients (65%) were men, while 28 patients (35%) were women. Out of them, 18 patients (22.5%) had diabetes, 14 (17.5%) patients had a smoking history, 12 (15%) patients had hypertension, and previous infections were present in nine (11.2%) patients. Twenty-seven patients (33.8%) had no comorbidities (Table 1).

**Table 1:** Demographic details of patients. The values are presented as N (%) where N is the number of patients.

Variable	Number (%)
Age (years)	Mean 42.5±12.4
Gender: Male	52 (65%)
Gender: Female	28 (35%)
Diabetes	18 (22.5%)
Smoking	14 (17.5%)
Hypertension	12 (15%)
Previous Infection	9 (11.2%)
No Comorbidities	27 (33.8%)

The femur was the most commonly affected bone (30, 37.5%), followed by the tibia (28, 35%), humerus (12, 15%), and radius/ulna (10, 12.5%). Open fractures occurred in 22 patients (27.5%), while 58 patients (72.5%) sustained closed fractures. Among open fractures, eight (10%) were Gustilo-Anderson type I, 10 (12.5%) type II, and four (5%) type III. Atrophic nonunion was observed in 38 patients (47.5%), hypertrophic in 28 (35%), and oligotrophic in 14 (17.5%). Of the 58 closed fractures, 26 (44.8%) were hypertrophic and 22 (37.9%) atrophic. In open fractures, 16 patients (72.7%) showed atrophic nonunion compared to only two (9%) with hypertrophic nonunion. Atrophic nonunion was more frequent in patients with comorbidities (28 of 53 patients, 52.8%) than in those without comorbidities (10 of 27 patients, 37%) (Table 2).

**Table 2:** Fracture characteristics The values are presented as N (%), where N is the number of patients.

Variable	Number (%)
Femur	30 (37.5%)
Tibia	28 (35%)
Humerus	12 (15%)
Radius/Ulna	10 (12.5%)
Open Fractures	22 (27.5%)
Closed Fractures	58 (72.5%)
Gustilo Type I (Open)	8 (10%)
Gustilo Type II (Open)	10 (12.5%)
Gustilo Type III (Open)	4 (5%)
Hypertrophic Nonunion	28 (35%)
Atrophic Nonunion	38 (47.5%)
Oligotrophic Nonunion	14 (17.5%)

Histopathological examination revealed viable bone in 24 patients (30%), fibrous tissue in 18 patients (22.5%), fibrocartilage in 12 patients (15%), necrotic bone in 14 patients (17.5%), and chronic inflammation in 12 patients (15%). Microbiological culture was positive in 14 patients (17.5%) and negative in 66 patients (82.5%) (Table 3).

**Table 3:** Histopathological and culture charecteristics The values are presented as N (%), where N is the number of patients.

Variable	Number (%)
Viable Bone	24 (30%)
Fibrous Tissue	18 (22.5%)
Fibrocartilage	12 (15%)
Necrotic Bone	14 (17.5%)
Chronic Inflammation	12 (15%)
Culture Positive	14 (17.5%)
Culture Negative	66 (82.5%)

Of the 24 patients with viable bone, 20 (83.3%) achieved complete healing, three (12.5%) showed partial healing, and only one (4.2%) had persistent nonunion. In contrast, among the 14 patients with necrotic bone, only four (28.5%) healed completely, five (35.7%) healed partially, and five (35.7%) had persistent nonunion. Culture-negative cases demonstrated a higher complete healing rate (46 of 66 patients, 69.7%) compared to culture-positive cases (four of 14 patients, 28.6%). These findings highlight the strong association of viable bone histology and negative culture status with improved healing outcomes, while necrotic bone and positive culture status were linked to poorer healing (Table 4).

**Table 4:** Healing outcome of patients The values are presented as N (%), where N is the number of patients.

Outcome	Number (%)
Completely Healed	50 (62.5%)
Partially Healed	18 (22.5%)
Persistent Non-union	12 (15%)

The correlation analysis between histopathological findings and healing outcomes at six months post-revision surgery showed that patients with viable bone had the highest complete healing rate (83.3%) and the lowest persistent nonunion rate (4.2%). This was statistically significant with a p-value of 0.05. Patients with necrotic bone had the highest persistent nonunion rate (35.7%) and the lowest complete healing rate (28.5%). Patients with fibrous tissue, fibrocartilage, and chronic inflammation had intermediate outcomes, with complete healing rates ranging from 33.3% to 55.5%. However, the p-values for these categories were all greater than 0.05, indicating that the differences were not statistically significant when compared with viable bone (Table 5). Thus, viable bone is a strong predictor for complete healing, whereas necrotic bone is most strongly associated with poor healing.

**Table 5:** Correlation between histopathological results and outcome of healing The values are presented as N (%), where N is the number of patients. p-value less the 0.05 is considered as significant.

Histopathology	Completely Healed n (%)	Partially Healed n (%)	Persistent Non-union n (%)	Chi-square value	p-value
Viable Bone (n=24)	20 (83.3%)	3 (12.5%)	1 (4.2%)	7.82	0.05*
Fibrous Tissue (n=18)	10 (55.5%)	6 (33.3%)	2 (11.1%)	1.69	0.607
Necrotic Bone (n=14)	4 (28.5%)	5 (35.7%)	5 (35.7%)	9.85	0.007*
Chronic Inflammation (n=12)	4 (33.3%)	5 (41.7%)	3 (25%)	3.42	0.061

The correlation between radiological classification and histopathological findings showed that hypertrophic nonunions had the highest proportion of viable bone (57.1%), suggesting good biological activity. Atrophic nonunions had a significantly higher proportion of necrotic bone (31.6%) and chronic inflammation (26.3%) compared to hypertrophic cases. Oligotrophic nonunions demonstrated an almost equal distribution of fibrous tissue, necrotic bone, and chronic inflammation (each 28.6%) (Table 6). The differences between radiological types were significant, with a p-value of 0.05 for hypertrophic and 0.04 for atrophic nonunions, indicating that radiological classification strongly correlates with biological activity at the nonunion site.

**Table 6:** Correlation between radiological type of non-union and histopathology The values are presented as N(%), where N is the number of patients. p-value less the 0.05 is considered as significant.

Radiological Type	Viable Bone n (%)	Fibrous Tissue n (%)	Necrotic Bone n (%)	Chronic Inflammation n (%)	Chi-square value	p-value
Hypertrophic (n=28)	16 (57.1%)	6 (21.4%)	2 (7.1%)	4 (14.3%)	8.42	0.05*
Atrophic (n=38)	6 (15.8%)	10 (26.3%)	12 (31.6%)	10 (26.3%)	9.15	0.04*
Oligotrophic (n=14)	2 (14.3%)	4 (28.6%)	4 (28.6%)	4 (28.6%)	4.73	0.106

The correlation of clinical and demographic factors with histopathological findings demonstrated that patients with diabetes had the highest proportion of necrotic bone (33.3%) and the lowest proportion of viable bone (27.8%). Similarly, patients with previous infections and hypertension also showed a high proportion of necrotic bone (33.3% and 25%, respectively). Patients with a smoking history had comparable proportions of viable bone (28.6%) and necrotic bone (28.6%), but a higher proportion of fibrous/fibrocartilage (42.8%). Patients without comorbidities had the most favorable profile, with the highest proportion of viable bone (37%) and the lowest necrotic bone (14.8%). The association between diabetes and adverse histopathological findings was statistically significant (p=0.03). Other comorbidities including smoking, hypertension, and previous infections showed p-values above 0.05, suggesting that their association with necrotic bone or adverse histopathology was not statistically significant (Table 7). This indicates that diabetes is the strongest predictor among comorbidities for poor biological activity at the nonunion site, whereas the absence of comorbidities was associated with better histopathological outcomes.

**Table 7:** Correlation between comorbidities and histopathology of non-union site The values are presented as N (%), where N is the number of patients. p-value less the 0.05 is considered as significant.

Factor	Viable Bone, n (%)	Necrotic Bone, n (%)	Fibrous/Fibrocartilage, n (%)	Chi-square value	p-value
Diabetes (n=18)	5 (27.8%)	6 (33.3%)	7 (38.9%)	7.21	0.03*
Smoking (n=14)	4 (28.6%)	4 (28.6%)	6 (42.8%)	3.94	0.09
Hypertension (n=12)	3 (25%)	4 (33.3%)	5 (41.7%)	3.56	0.07
Previous infections (n=11)	3 (27.3%)	3 (27.3%)	5 (45.4%)	2.92	0.15
No comorbidities (n=27)	10 (37%)	4 (14.8%)	13 (48.2%)	6.11	0.05*

Culture-negative patients had a higher complete healing rate (69.7%) and a lower persistent nonunion rate (10.6%). Culture-positive patients had a much lower complete healing rate (28.6%) and a persistent non-union rate of 35.7% (Table 8). This difference remained statistically significant, showing that infection at the non-union site negatively impacts the likelihood of healing.

**Table 8:** Correlation between microbiological status and healing outcomes. The values are presented as N(%), where N is the number of patients. p-value less the 0.05 is considered as significant. Fischer's Exact test is used here for calculating the p-value.

Microbiological Status	Completely Healed, N (%)	Persistent Non-union, N (%)	Odds Ratio (OR)	p-value
Culture Negative (n=66)	46 (69.7%)	7 (10.6%)	6.57	<0.001
Culture Positive (n=14)	4 (28.6%)	5 (35.7%)	0.80	1.000

## Discussion

Fracture nonunion remains a major clinical challenge in orthopedic trauma, affecting 2%-10% of long-bone fractures and leading to prolonged disability, impaired function, and increased healthcare costs [1]. In our cohort, the persistent nonunion rate at six months post-revision surgery was 15%, which aligns with the upper range of previously reported data [1]. Several well-established risk factors contribute to nonunion, including diabetes mellitus, smoking, open fractures, and infection, all of which impair vascularity and disrupt the normal healing cascade [2]. Our findings corroborate these observations: diabetes was significantly associated with necrotic bone histology and poorer healing outcomes (p=0.03), which is consistent with prior studies identifying diabetes as a major predictor of impaired bone regeneration [2]. Smoking and infection also trended toward poorer healing in our cohort, though these associations did not reach statistical significance, a finding similar to smaller observational studies [2,3].

Histopathological studies have consistently demonstrated that nonunion tissue comprises a broad spectrum of elements, including viable woven bone, fibrocartilage, fibrous tissue, necrotic bone, and chronic inflammatory infiltrates [4]. Our analysis reflected this distribution, with viable bone observed in 30% of cases, fibrous/fibrocartilage in 37.5%, necrotic bone in 17.5%, and chronic inflammation in 15%. This closely mirrors the proportions described in previous histopathological surveys [4]. Importantly, viable bone histology strongly correlated with favorable healing outcomes, while necrotic bone and chronic inflammation were predictive of persistent nonunion, echoing findings from prior clinical-histological studies [4]. In our cohort, 83.3% of patients with viable bone achieved complete healing compared with only 28.5% of those with necrotic bone (p=0.04). These results are comparable to the predictive associations reported in large multicenter datasets [4].

Radiological classification into hypertrophic, atrophic, and oligotrophic nonunions has traditionally been used to estimate the biological activity of a fracture site, with hypertrophic types indicating preserved healing potential but insufficient stability, and atrophic types suggesting impaired vascularity and cellular activity [5]. However, several studies have demonstrated that radiological appearance alone cannot reliably predict the underlying biological viability, and histopathological evaluation is often required for a more accurate assessment [5,6]. Our findings support this observation, as several radiologically atrophic cases in our cohort demonstrated viable bone or fibrocartilage on histology, while some hypertrophic cases contained necrotic bone, illustrating the discrepancy between imaging and the actual biological environment. This discrepancy is further highlighted by studies showing that there is no significant difference in vessel density between atrophic and hypertrophic nonunions, challenging the classical belief that atrophic nonunions are entirely avascular [6]. Similarly, studies in scaphoid nonunions have shown that MRI findings, particularly low T1 signal intensity, correlate more closely with histological necrosis than CT imaging, which can miss subtle areas of osteonecrosis [7]. Our results paralleled these findings, as we identified cases radiologically classified as “stable” hypertrophic nonunions that harbored substantial necrotic bone or extensive fibrosis, which could compromise healing if not addressed appropriately.

Systemic comorbidities, such as age, diabetes, and prior infection, have been reported to influence the radiological appearance and biological potential of nonunions [8]. In our study, diabetes and previous infection were significantly associated with necrotic bone histology (p=0.03 and 0.06, respectively), particularly among radiologically atrophic cases. These findings are consistent with previous reports that systemic factors can adversely affect the biological milieu at nonunion sites [8]. Furthermore, there is growing evidence that atrophic nonunions may still harbor viable mesenchymal stromal cells and osteoprogenitors, suggesting that their biological potential may not be completely absent [9]. We observed viable bone or fibrous/fibrocartilage in over 60% of radiologically atrophic nonunions, supporting the notion that biological augmentation combined with mechanical stabilization may lead to successful union in these challenging cases.

The integration of microbiological and histopathological findings provides additional prognostic value in predicting healing outcomes in nonunion cases. Culture-positive infections at the nonunion site have been consistently associated with poorer biological activity and lower rates of successful union [10]. In our cohort, patients with culture-positive nonunions demonstrated a significantly lower complete healing rate (28.6%) compared to culture-negative cases (69.7%), a finding that aligns with previously published studies, highlighting the adverse effect of infection on bone healing [10]. Chronic low-grade infections may lead to persistent inflammation and necrosis, as reflected by the higher proportion of necrotic bone and chronic inflammatory infiltrates in infected cases observed in our study.

Our findings also reinforce the importance of comorbidity control and host optimization prior to surgical intervention. Diabetes, smoking, and previous infection were all associated with unfavorable histopathological profiles and poorer outcomes, although only diabetes reached statistical significance in our analysis. These results are consistent with literature demonstrating that systemic comorbidities not only impair vascularity and osteogenesis but also increase the susceptibility to persistent infection at the fracture site [2,8]. Therefore, targeted preoperative optimization, such as strict glycemic control and smoking cessation, may help improve biological healing potential. From a treatment perspective, our data support the use of biological augmentation strategies, including autologous bone grafting and adjuvants such as bone morphogenetic proteins (BMPs), particularly in cases with histological evidence of necrotic bone or extensive fibrosis [11,12]. Several studies have shown that BMPs and other biologics can stimulate angiogenesis and osteogenesis, improving the likelihood of union in biologically compromised cases [11,12]. Moreover, because radiological classification alone may fail to capture the true biological status, we recommend that intraoperative histopathological assessment be considered in difficult cases to better tailor management.

In a recent systematic review and meta-analysis [13], surprise positive cultures were identified in approximately 16% of patients with presumed aseptic long-bone nonunions, and these cases were associated with significantly higher rates of secondary surgery and poorer outcomes. While these multicenter data highlight the clinical relevance of occult infection, our study differs by prospectively integrating microbiological culture results with histopathological evaluation of the nonunion tissue and directly correlating both with short-term healing outcomes. We demonstrated that culture-positive cases not only had significantly lower healing rates but also showed higher proportions of necrotic bone and chronic inflammation on histology, thereby linking microbiological findings with underlying biological compromise. This comprehensive correlation is not addressed in most prior reviews and provides a unique contribution by underscoring the combined prognostic value of histopathology and culture in predicting the biological potential for union.

Limitations of the study

Our study has certain limitations that must be acknowledged. The relatively small sample size and single-center design may limit the generalizability of the findings. In addition, histopathological assessment was performed by a single pathologist without inter-observer validation, which could introduce observer bias. The follow-up period was restricted to six months, thereby limiting the evaluation of long-term healing and refracture risk. Furthermore, functional outcomes such as pain, mobility, and return to activity were not systematically assessed, and thus the correlation of histopathological and microbiological findings with functional recovery could not be established. Future multicenter studies with longer follow-up, blinded multi-observer histopathology review, and inclusion of standardized functional outcome measures are warranted to validate and extend our observations. Some findings in our study have borderline p-values, so claims should be stated more cautiously. Such studies may ultimately lead to the development of integrated algorithms combining clinical, radiological, microbiological, and histological data to guide personalized treatment strategies [4,14-16].

## Conclusions

Fracture nonunion remains a multifactorial condition with significant clinical and socioeconomic impact. Our findings highlight that histopathological evaluation adds valuable biological information beyond radiological classification, enabling better prediction of healing potential. Viable bone histology was strongly associated with successful healing, whereas necrotic bone and chronic inflammation predicted persistent nonunion. Comorbidities such as diabetes and previous infection adversely influenced histological profiles and outcomes, reinforcing the importance of preoperative optimization. Additionally, culture-positive nonunions demonstrated lower healing rates, underscoring the need for meticulous infection control. A combined approach that integrates clinical risk factors, radiological appearance, microbiological status, and histopathology can guide individualized treatment strategies, particularly identifying cases that may benefit from biological augmentation. Further multicenter studies are required to validate these observations and establish standardized algorithms for the evaluation and management of nonunion.
